# Duration of Anti-Tuberculosis Therapy and Timing of Antiretroviral Therapy Initiation: Association with Mortality in HIV-Related Tuberculosis

**DOI:** 10.1371/journal.pone.0074057

**Published:** 2013-09-16

**Authors:** Claudia P. Cortes, Firas H. Wehbe, Catherine C. McGowan, Bryan E. Shepherd, Stephany N. Duda, Cathy A. Jenkins, Elsa Gonzalez, Gabriela Carriquiry, Mauro Schechter, Denis Padgett, Carina Cesar, Juan Sierra Madero, Jean W. Pape, Daniel R. Masys, Timothy R. Sterling, and the Caribbean, Central American, South American Network for HIV Research (CCASA-net) of the International Epidemiologic Databases to Evaluate AIDS (IeDEA)

**Affiliations:** 1 University of Chile and Fundación Arriarán, Santiago, Chile; 2 Vanderbilt University, Nashville, Tennessee, United States of America; 3 Instituto de Medicina Tropical Alexander von Humboldt, Universidad Peruana Cayetano Heredia, Lima, Perú; 4 Universidad Federal do Rio de Janeiro, Rio de Janeiro, Brazil; 5 Instituto Hondureño de Seguro Social y Hospital Escuela, Tegucigalpa, Honduras; 6 Fundación Huesped, Buenos Aires, Argentina; 7 Instituto Nacional de Ciencias Medicas y Nutrición Salvador Zubirán, Mexico City, Mexico; 8 Les Centres GHESKIO, Port au Prince, Haiti; University of New South Wales, Australia

## Abstract

**Background:**

Antiretroviral therapy (ART) decreases mortality risk in HIV-infected tuberculosis patients, but the effect of the duration of anti-tuberculosis therapy and timing of anti-tuberculosis therapy initiation in relation to ART initiation on mortality, is unclear.

**Methods:**

We conducted a retrospective observational multi-center cohort study among HIV-infected persons concomitantly treated with Rifamycin-based anti-tuberculosis therapy and ART in Latin America. The study population included persons for whom 6 months of anti-tuberculosis therapy is recommended.

**Results:**

Of 253 patients who met inclusion criteria, median CD4+ lymphocyte count at ART initiation was 64 cells/mm^3^, 171 (68%) received >180 days of anti-tuberculosis therapy, 168 (66%) initiated anti-tuberculosis therapy before ART, and 43 (17%) died. In a multivariate Cox proportional hazards model that adjusted for CD4+ lymphocytes and HIV-1 RNA, tuberculosis diagnosed after ART initiation was associated with an increased risk of death compared to tuberculosis diagnosis before ART initiation (HR 2.40; 95% CI 1.15, 5.02; P = 0.02). In a separate model among patients surviving >6 months after tuberculosis diagnosis, after adjusting for CD4+ lymphocytes, HIV-1 RNA, and timing of ART initiation relative to tuberculosis diagnosis, receipt of >6 months of anti-tuberculosis therapy was associated with a decreased risk of death (HR 0.23; 95% CI 0.08, 0.66; P=0.007).

**Conclusions:**

The increased risk of death among persons diagnosed with tuberculosis after ART initiation highlights the importance of screening for tuberculosis before ART initiation. The decreased risk of death among persons receiving > 6 months of anti-tuberculosis therapy suggests that current anti-tuberculosis treatment duration guidelines should be re-evaluated.

## Introduction

According to 2010 World Health Organization estimates, 1.7 million people in Latin America and the Caribbean are living with HIV/AIDS. Among HIV-infected persons, tuberculosis is one of the leading opportunistic infections and causes of death. Of the estimated 8.7 million incident tuberculosis cases globally in 2011, up to 13% occurred in HIV-infected persons, leading to 430,000 deaths among HIV- infected tuberculosis patients [1]. Of these deaths, 37,000 occurred in the Americas [1].

Antiretroviral therapy (ART) significantly improves survival among HIV-infected tuberculosis patients [2,3,4]. Among HIV-infected persons who develop tuberculosis before starting ART and have < 50 CD4+ lymphocytes/mm^3^ at the time of tuberculosis diagnosis, starting of ART within 2-4 weeks of initiating anti-tuberculosis therapy significantly improves survival [5,6,7]. However, among HIV-infected persons who develop tuberculosis after initiating ART, persons diagnosed with tuberculosis in the first 3 months of ART may have an increased risk of death compared to those who develop tuberculosis later [8,9]. It is unclear whether the risk of death for those diagnosed in the first 3 months of ART is higher than among persons who are diagnosed with tuberculosis before starting ART [10,11]. The optimal duration of anti-tuberculosis therapy among HIV-infected persons is also unclear. Although current recommendations are to treat pulmonary tuberculosis with rifamycin-based therapy for six months [12,13,14]—the same as in HIV-uninfected persons—recent data suggest that tuberculosis relapse rates may be unacceptably high in HIV-infected persons treated for six months [15,16,17,18]. . In fact, some clinicians and programs treat for more than six months [19,20,16].

We therefore assessed the mortality rate among HIV-infected tuberculosis patients who concomitantly received ART—initiated either before or after tuberculosis diagnosis. We also assessed the relationship between duration of anti-tuberculosis therapy and the risk of death, to inform the optimal duration of anti-tuberculosis treatment.

## Methods

We conducted a retrospective observational cohort study within the Caribbean, Central and South America Network for HIV Research (CCASAnet) of the International Epidemiologic Databases to Evaluate AIDS (IeDEA; www.iedea.org) [21]. The core dataset was composed of observational databases from six participating HIV clinics in Argentina, Brazil, Chile, Honduras, Mexico, and Peru. Additional tuberculosis data were collected using REDCap, a secure, web-based, electronic case report form system hosted at Vanderbilt University. Institutional review board approval for the study was obtained at each site and at the CCASAnet data coordinating center at Vanderbilt.

The cohort included HIV-infected adults (> 18 years old) who initiated ART between 1997 and 2007 and were followed for up to one year or more after ART initiation. Patients were included if they received ART at any time while concomitantly receiving rifamycin-based (i.e., rifampin or rifabutin) anti- tuberculosis therapy. ART could have been started before or after anti-tuberculosis therapy initiation. To assess the effect of duration of anti-tuberculosis therapy on mortality, we limited the study to persons for whom 6 months of therapy was recommended (e.g., those with pulmonary, lymphatic, pleural disease). Persons with central nervous system, bone, joint, or pericardial tuberculosis were excluded because recommendations indicate at least 9-12 months of therapy [12].

### Study definitions

ART was defined as regimens that included three or more antiretroviral drugs. For this study, the sentinel episode of tuberculosis was the participant’s first tuberculosis episode during which they concomitantly received ART. Tuberculosis diagnoses were established by each study site, based on smear, culture, clinical and radiographic criteria, as well as response to anti-tuberculosis therapy. The date of tuberculosis diagnosis was defined as the date of anti-tuberculosis therapy initiation. Tuberculosis recurrence was defined as development of a new tuberculosis case after completing treatment of the sentinel episode. All tuberculosis cases were validated by review of the participant’s medical records and primary source documentation by local site investigators; independent quality control and TB case validation were performed at each study site (CCM, SND). Baseline CD4+ lymphocyte and HIV-1 RNA measurements were defined as at the time of ART initiation; measurements prior to tuberculosis diagnosis were also utilized. Baseline CD4+ lymphocyte count was defined as the measurement closest to ART initiation but not more than 6 months prior to, or 7 days after, the date of ART start. Baseline HIV-1 RNA was defined as the pre-ART measurement closest to, but not more than 6 months prior to ART initiation. The same window periods were used for measurements prior to tuberculosis diagnosis. Episodes of immune reconstitution inflammatory syndrome (IRIS) were captured, as defined by local site investigators.

### Statistical analysis

Groups were defined according to timing of ART initiation in relation to anti-tuberculosis therapy initiation. Continuous variables were compared with the Wilcoxon rank-sum test. Categorical variables were compared with the Pearson chi-square test. For the evaluation of risk factors for death after tuberculosis diagnosis, univariate and multivariate Cox proportional hazards models stratified by site were constructed. Variables in the multivariate models were determined a priori based on their clinical relevance regarding survival. Missing CD4+ lymphocyte and HIV-1 RNA values were imputed via multiple imputation for multivariable models. For the evaluation of anti-tuberculosis treatment duration and mortality, the analysis was performed among a) all persons with known anti-tuberculosis therapy duration regardless of duration, b) limited to persons with known therapy duration who survived at least 6 months from initiation of treatment for the sentinel TB episode, and c) limited to persons with known therapy duration who survived at least seven months to compare persons who received 5-7 months vs. > 7 months of therapy. Deaths occurring before or after one year of follow-up from time of tuberculosis diagnosis were included. P-values < 0.05 were considered statistically significant.

## Results

Of the 3,539 HIV- infected persons who initiated ART during the study period, 533 (15%) received a diagnosis of tuberculosis. We excluded 219 patients who did not receive concomitant anti-tuberculosis therapy and ART, 20 patients who did not receive rifamycin-based anti-tuberculosis therapy, 40 patients who required extended TB treatment (e.g., due to tuberculosis of the central nervous system, bone / joint, or pericardium), and 1 patient with 0 days of follow-up. The final TB dataset included 253 patients who met all inclusion criteria. The clinical and demographic characteristics of the study population are in Table 1. Persons diagnosed with tuberculosis were younger, more likely to be from Peru, have heterosexual sex as their HIV risk factor, have lower CD4+ lymphocyte count and higher HIV-1 RNA at ART initiation, and more likely to die than persons not diagnosed with tuberculosis. Tuberculosis patients also tended to more likely be male.

**Table 1:** Characteristics of the Study Population.

**Characteristic**	**No TB N = 3,006**	**TB N = 253**	**P-value**
Median age (IQR)	36 (30-43)	35 (28-40)	0.002
Male sex	2,195 (73)	198 (78)	0.07
Study site*			<0.001
Argentina	769 (26)	22 (9)	
Brazil	468 (16)	34 (13)	
Chile	518 (17)	20 (8)	
Honduras	286 (10)	28 (11)	
Mexico	397 (13)	16 (6)	
Peru	568 (19)	133 (53)	
HIV risk factor			0.03
Heterosexual	1,433 (48)	146 (58)	
MSM	1,098 (37)	80 (32)	
IDU	80 (3)	6 (2)	
Other	25 (1)	1 (0.4)	
Unknown	370 (12%)	20 (8)	
Baseline median CD4 (IQR)	119 (44-210) N=2,449	64 (26-141)^ N=204	<0.001
Baseline median HIV-1 RNA in log_10_ (IQR)	4.9 (4.6-5.4) N=1,796	5.2 (4.9-5.6)# N=134	<0.001
Death	199 (7)	43 (17)	<0.001

HIV-infected persons who initiated antiretroviral therapy (ART), received rifamycin-based anti-tuberculosis therapy while on ART, and did not have tuberculosis at a site of disease that required extended therapy (e g, central nervous system, bone/joint, or pericardium).

Parentheses include percentages unless otherwise noted.

TB: tuberculosis

Baseline CD4+ lymphocyte and HIV-1 RNA measurements were at the time of ART initiation.

* The proportions of all persons at each study site with TB (the row percentages)—were as follows:

Argentina: 3%; Brazil: 7%; Chile: 4%; Honduras: 9%; Mexico: 4%; Peru: 16%

^ Median (IQR) values before tuberculosis diagnosis: 74 (34-191) # Median (IQR values before tuberculosis diagnosis: 4.4 log_10_ (2.6-5.2)

Characteristics of the 253 tuberculosis cases are listed in Table 2 according to length of follow-up/survival and duration of anti-tuberculosis therapy. Most patients had pulmonary disease as well as extrapulmonary disease. Approximately one-quarter were culture-confirmed and less than half were acid fast smear-positive. There were 168 (66%) tuberculosis cases diagnosed before ART was initiated, and approximately 90% of the patients received NNRTI-based ART. There were 171 (68%) patients treated for 180 days, and almost all received daily therapy during the first two months of treatment. Of the 253 sentinel tuberculosis cases, 15 (6%) subsequently had recurrent disease. Of the 20 tuberculosis cases in Chile, 1 (5%) developed recurrence, and of the 133 tuberculosis cases in Peru, 14 (11%) developed recurrence; there were no recurrences reported at the other study sites.

**Table 2:** Baseline and Treatment Characteristics of the 253 Tuberculosis Patients Included in the Study, According to Length of Follow-up/Survival and Duration of Anti-tuberculosis Therapy.

**Characteristic**	**< 6 months of follow-up and TB treatment**	**> 6 months of follow-up after TB diagnosis**		**> 6 months of follow-up; TB treatment duration unknown**	
	**N=25**	**TB treatment < 6 months N= 29**	**TB treatment > 6 months N= 171**	**N=28**	**P-value**
Median age (IQR)	35 (31, 39)	31 (27, 38)	35 (28, 41)	36 (26, 38)	0.83
Male sex	21 (84)	22 (76)	133 (78)	22 (79)	0.90
HIV risk factor					0.81
Heterosexual	14 (56)	17 (59)	97 (57)	18 (64)	
MSM	10 (40)	11 (38)	50 (29)	9 (32)	
IDU	0 (0)	0 (0)	6 (4)	0 (0)	
Other/unknown	1 (4)	1 (3)	18 (11)	1 (4)	
Baseline CD4 (IQR)	53 (26, 137)	78 (44, 189)	62 (23, 142)	85 (38, 128)	0.78
Baseline HIV-1 RNA log_10_ (IQR)	5.0 (4.8, 5.3)	5.3 (4.9, 5.4)	5.1 (4.9, 5.6)	5.6 (5.0, 5.8)	0.20
Site of disease					
Pulmonary	20 (80)	22 (76)	132 (77)	24 (86)	0.62
Extrapulmonary	10 (42)	17 (59)	85 (50)	10 (37)	0.48
Culture-positive	8 (32)	9 (31)	46 (27)	4 (14)	0.41
Smear-positive	9 (36)	9 (31)	76 (44)	15 (54)	0.31
Time of TB Diagnosis					<0.001*
Before ART Start^a^	9 (36)	18 (62)	127 (74)	14 (50)	
After ART Start^b^	16 (64)	11 (38)	44 (26)	14 (50)	
History of Prior TB	0 (0)	2 (7)	6 (4)	3 (11)	0.20
Type of ART					0.47
PI-based	3 (12)	1 (3)	21 (12)	0 (0)	
NNRTI-based	22 (88)	28 (97)	148 (87)	28 (100)	
Efavirenz	11 (44)	14 (48)	90 (53)	17 (61)	
Nevirapine	11 (44)	14 (48)	58 (34)	11 (39)	
Triple NRTI	0 (0)	0 (0)	2 (1)	0 (0)	
TB treatment duration^=^ Median in days (IQR)	46 (18, 104)	132 (82, 173)	271 (236, 352)	—	<0.001
First 2 months of treatment^^^					0.24
Daily TB treatment	23 (100)	25 (96)	151 (99)	21 (100)	
Intermittent TB Rx	0 (0)	0 (0)	1 (1)	0 (0)	
Unknown	0 (0)	1 (4)	0 (0)	0 (0)	
Continuation phase^#^					<0.001
Daily (5-7 days)	2 (22)	4 (19)	53 (41)	1 (9)	
Three times/week	1 (11)	4 (19)	40 (31)	2 (18)	
Two times/week	5 (55)	11 (52)	34 (26)	1 (9)	
Unknown	1 (11)	2 (10)	3 (2)	7 (64)	
Immune Reconstitution Inflammatory Syndrome (IRIS)	0 (0)	2 (7)	0 (0)	9 (5)	0.34
Post-treatment INH^+^					<0.001*
Yes	0 (0)	1 (4)	5 (3)	0 (0)	
No	18 (86)	19 (76)	133 (82)	7 (35)	
Unknown	3 (14)	5 (20)	24 (15)	13 (65)	
Follow-up duration Median in months (IQR)	2.5 (1.1, 3.5)	36.4 (17.3, 64.1)	48.3 (33.8, 63.4)	27.8 (18.2, 45.2)	<0.001
TB recurrence	0 (0)	5 (17)	7 (4)	3 (11)	0.02
Death (after TB diagnosis)	19 (76)	6 (21)	13 (8)	5 (18)	<0.001

Data are presented as number (%) except as shown.

P-values are for the comparison of all 4 groups, using the chi-squared test. * the comparison limited to persons with > 6 months of follow-up and known TB treatment duration (the two inner columns) was not statistically significant. If not noted by * the statistical significance of the comparison between these two groups was similar to that of all 4 groups.

=Data available for 222 patients.

^ Data available for 222 patients. # Data available for 171 patients. + Data available for 228 patients. a median time between TB diagnosis and ART start: 95 days (IQR: 53, 142) b median time between ART start and TB diagnosis: 142 days (IQR: 30, 568)

There were no statistically significant differences in age, sex, HIV risk factor, CD4+ lymphocyte count at ART initiation or before tuberculosis diagnosis, HIV-1 RNA at ART initiation or before tuberculosis diagnosis, or death between those who did vs. did not develop recurrent tuberculosis (data not shown). Compared to persons with tuberculosis who survived, those who died had lower median CD4+ lymphocyte count at ART initiation (45 vs. 66 cells/mm^3^; P=0.02) and before tuberculosis diagnosis (59 vs. 85 cells/mm^3^; P=0.01). They also tended to more likely be female and from Peru. There was no statistically significant difference in age, smear-negative disease, HIV risk factor, or HIV-1 RNA at ART initiation or before tuberculosis diagnosis.

In univariate Cox proportional hazards models of time from tuberculosis diagnosis to death among all tuberculosis patients, being diagnosed with tuberculosis after ART initiation tended to be associated with an increased risk of death compared to persons diagnosed with tuberculosis before ART initiation (Table 3). The findings were similar (but achieved statistical significance) in the multivariable model: patients on ART when tuberculosis was diagnosed had a higher rate of death than those diagnosed with tuberculosis prior to ART initiation, after adjusting for CD4+ lymphocyte count and HIV-1 RNA. Results were similar after adjusting for era of ART initiation (1998-2001 vs. 2002-2007), and did not differ statistically between eras (data not shown). An additional multivariable model demonstrated that patients diagnosed with tuberculosis <90 days after ART initiation had a similarly increased risk of death as those diagnosed > 90 days after ART initiation (both compared to persons with tuberculosis diagnosed before ART initiation (< 90 days: aHR 2.44 (95% CI: 0.93, 6.39; P = 0.07; > 90 days: aHR 2.38 (95% CI: 1.01, 5.6; P = 0.05).

**Table 3:** Risk Factors for Death Among All Tuberculosis Patients.

**Characteristic**	**Univariate**			**Multivariate**		
	HR	95% CI	P-value	HR	95% CI	P-value
CD4 before TB (per 100 ↑)	0.70	0.46, 1.07	0.10	0.66	0.43, 1.01	0.06
HIV-1 RNA before TB (log_10_ transformed)	1.01	0.74, 1.37	0.97	1.03	0.75, 1.42	0.85
On ART when TB diagnosed*	1.83	0.95, 3.51	0.07	2.40	1.15, 5.02	0.02

Cox proportional hazards models of time to death. The models are stratified by study site. Follow-up is from the time of tuberculosis diagnosis. For the multivariable models, missing CD4+ lymphocyte and HIV-1 RNA values were imputed via multiple imputation. Of the 222 subjects, 44 had missing CD4+ lymphocytes and 59 had missing HIV- 1 RNA.

* Compared to persons starting ART after TB diagnosis

Among tuberculosis patients who survived at least 6 months from the time of tuberculosis diagnosis, receipt of > 6 months of anti-tuberculosis therapy was associated with a significantly decreased risk of death after adjusting for CD4+ lymphocyte count and HIV-1 RNA at the time of tuberculosis diagnosis, as well as timing of ART initiation in relation to anti-tuberculosis therapy initiation (HR 0.23; 95% CI 0.08, 0.66; P=0.007; Table 4).

**Table 4:** Risk Factors for Death Among Tuberculosis Patients Surviving at Least 6 Months from Tuberculosis Diagnosis.

**Characteristic**	**Univariate**			**Multivariate**		
	HR	95% CI	P-value	HR	95% CI	P-value
CD4 before TB (per 100 ↑)	0.79	0.46, 1.34	0.38	0.65	0.35, 1.20	0.17
HIV-1 RNA before TB (log_10_ transformed)	1.22	0.65, 1.82	0.41	1.23	0.75, 2.00	0.41
On ART when TB diagnosed*	0.96	0.34, 2.68	0.93	1.33	0.41, 4.28	0.63
>6 months of TB treatment	0.30	0.11, 0.81	0.02	0.23	0.08, 0.66	0.007

Cox proportional hazards models of time to death. The models are stratified by study site. Follow-up is from the time of tuberculosis diagnosis. For the multivariable models, missing CD4+ lymphocyte and HIV-1 RNA values were imputed via multiple imputation.

* Compared to persons starting ART after TB diagnosis

There were 200 persons who survived at least 6 months, of whom 19 died.

Of the 200 persons, 40 had missing CD4+ lymphocytes and 53 had missing HIV-1 RNA.

The median duration of anti-tuberculosis treatment among persons surviving at least 6 months was 258 days (IQR: 193, 333)

Persons who received > 7 months of anti-tuberculosis treatment had a lower risk of death than persons who received 5-7 months of treatment after controlling for CD4+ lymphocyte count and HIV-1 RNA at the time of tuberculosis diagnosis, as well as timing of ART initiation in relation to anti-tuberculosis therapy initiation, but the difference was not statistically significant (aHR = 0.58; 95% CI 0.17, 1.94; P = 0.38).

Among the 153 patients who started ART after tuberculosis diagnosis whose tuberculosis treatment duration was known, 41 started within 60 days, 30 started within 61-90 days, 27 started within 91-120 days, 55 started >120 days after tuberculosis diagnosis. Patients who initiated ART >60 or >90 days after TB diagnosis had delayed progression from tuberculosis to death after adjusting for CD4+ lymphocyte count (Table 5). Results were similar among persons initiating ART >120 days after tuberculosis diagnosis, but they did not achieve statistical significance (Table 5).

**Table 5:** Risk Factors for Death According to Time of ART Start after Tuberculosis Diagnosis.

	>60 days			>90days			>120 days		
	HR	95% CI	P-value	HR	95% CI	P-value	HR	95% CI	P-value
Days from TB diagnosis	0.33	0.13, 0.83	0.02	0.29	0.11, 0.83	0.01	0.38	0.13, 1.09	0.07
CD4 before TB (per 100 ↑)	0.45	0.17, 1.15	0.10	0.51	0.22, 1.15	0.13	0.53	0.22, 1.28	0.16

Cox proportional hazards models. Comparator group is persons starting HAART within the specific timeframe (< 60, 90, or 120 days from TB diagnosis). Missing CD4+ lymphocyte values were imputed via multiple imputation.

Of the 153 persons who started ART after tuberculosis diagnosis, 41 started with 60 days, 71 started within 90 days, and 98 started within 120 days of TB diagnosis. Of the 153 persons, 31 had missing CD4+ lymphocytes.

## Discussion

There were several important findings of this study, related to the duration and timing of tuberculosis treatment in HIV-infected persons. First, patients who received more than six months of tuberculosis treatment had a significantly decreased risk of death compared to persons receiving less than six months. We are unaware of other studies that have assessed the effect of anti-tuberculosis therapy duration on mortality among HIV-infected tuberculosis patients who concomitantly received antiretroviral therapy. However, there have been several studies and a meta-analysis suggesting that more than six months of anti-tuberculosis therapy is associated with lower rates of tuberculosis treatment failure and relapse [17,22,19,20,16]. There has been one randomized, controlled trial of 6 vs. 9 months of anti-tuberculosis therapy in HIV-infected persons, and there was no significant difference in the risk of death [18]. However, only one-fifth of the study population had access to ART, and all patients received intermittent anti-tuberculosis therapy.

Although our study suggests that more than six months of anti-tuberculosis therapy is associated with improved survival, our analysis has limitations. To minimize bias, we performed analyses including only persons who survived at least 6 months after tuberculosis diagnosis, However, in clinical practice, not all persons assigned to at least 6 months of treatment would survive six months. Among persons who survived at least 6 months, the clinical and demographic characteristics of those treated for < 6 months vs. > 6 months were generally similar. However, those treated for < 6 months were also more likely to receive twice-weekly therapy in the continuation phase than those who received > 6 months; this could have contributed to the worse outcome in the former group. Furthermore, in an additional analysis comparing 5-7 months of anti-tuberculosis therapy with more than seven months of therapy among patients who survived at least 7 months, the association between prolonged therapy and improved survival weakened. In addition, longer treatment duration also has potential adverse effects given the drug-drug interactions with antiretroviral therapy and overlapping drug toxicity. These facts highlight the need for a large, prospective, randomized trial of two treatment durations among patients on ART. Study outcomes would include mortality and tuberculosis failure/relapse.

Another important finding of our study was the higher risk of death among persons diagnosed with tuberculosis after ART initiation compared to those diagnosed before ART initiation. This has also been observed in HIV-infected tuberculosis patients in Haiti [8], but was not seen in a similar patient population in Uganda [10]. The reasons for the increased risk of death are unclear, and may be related to our study inclusion criteria (concomitant tuberculosis treatment and ART) that excluded persons who rapidly progressed from tuberculosis diagnosis to death before ART initiation. The increased risk of death may also be related to unmasking of previously sub-clinical tuberculosis and paradoxical worsening associated with IRIS [23,24,25]. However, studies to date have shown that unmasked tuberculosis is not associated with higher mortality [26,27]. Few patients in this study were reported to have developed IRIS. The fact that the risk of death was lower among persons diagnosed with tuberculosis prior to ART initiation highlights the importance of screening for tuberculosis prior to initiating ART. In addition, persons not found to have tuberculosis before ART initiation should be closely monitored for signs and symptoms of tuberculosis, particularly during the first three month after ART initiation, so that treatment can be initiated promptly when tuberculosis is suspected.

ART started >60 or >90 days after anti-tuberculosis treatment initiation was associated with a decreased risk of death. In three recent randomized controlled trials, initiation of ART within 30 days of anti-tuberculosis therapy was associated with a decreased risk of AIDS and death, but only among persons with baseline CD4+ lymphocytes < 50 cells/mm^3^; the risk of disease progression and death tended to be increased among persons with higher CD4 counts [5,6,7]. The median CD4+ lymphocyte count at ART initiation among persons with tuberculosis in our study was 64/mm^3^. Since initiation of ART was an inclusion requirement for our study, tuberculosis patients who delayed ART such that they died before initiation were not included, and those classified as starting ART >60 or >90 days after anti-tuberculosis therapy were guaranteed to live >60 or >90 days, respectively, possibly biasing our results. Additional operational studies are warranted.

There were several additional limitations of our study. First, it was retrospective and observational. There may have been potential unmeasured confounding factors such as tuberculosis drug resistance, which affected the results. Second, the proportion of patients with culture-confirmed tuberculosis was low. Although this could potentially affect the generalizability of these findings to other settings, all patients met clinical criteria for tuberculosis and were diagnosed according to the standard of care in their respective countries. However, the low proportion of patients with culture-confirmed tuberculosis contributed to the lack of information on drug resistance. Resistance rates likely vary by study site. Third, while we captured data on IRIS, there was likely under-ascertainment of this endpoint given the retrospective study design. Fourth, the tuberculosis treatment duration may have included interruptions, which would have extended the overall duration. Finally, while directly-observed therapy (DOT) is recommended in all study sites, we did not have information on the number of patients who received DOT.

With the above limitations noted, we conclude that the decreased risk of death among persons receiving more than six months of anti-tuberculosis therapy suggests that current anti-tuberculosis treatment duration guidelines should be re-evaluated. A prospective, randomized trial is needed to determine the optimal anti-tuberculosis treatment duration among HIV-infected persons receiving ART. The increased risk of death among persons diagnosed with tuberculosis after ART initiation highlights the importance of screening for tuberculosis before ART initiation.
